# Role of Angiogenesis and Its Biomarkers in Development of Targeted Tumor Therapies

**DOI:** 10.1155/2024/9077926

**Published:** 2024-01-04

**Authors:** Anchal Pathak, Ajay Kumar Pal, Subhadeep Roy, Mukesh Nandave, Keerti Jain

**Affiliations:** ^1^Drug Delivery and Nanomedicine Research Laboratory, Department of Pharmaceutics, National Institute of Pharmaceutical Education and Research (NIPER)-Raebareli, Lucknow, India; ^2^Department of Pharmacology, Delhi Pharmaceutical Sciences and Research University (DPSRU), New Delhi 110017, India; ^3^Department of Pharmacology and Toxicology, National Institute of Pharmaceutical Education and Research, Kolkata, West Bengal, India

## Abstract

Angiogenesis plays a significant role in the human body, from wound healing to tumor progression. “Angiogenic switch” indicates a time-restricted event where the imbalance between pro- and antiangiogenic factors results in the transition from prevascular hyperplasia to outgrowing vascularized tumor, which eventually leads to the malignant cancer progression. In the last decade, molecular players, i.e., angiogenic biomarkers and underlying molecular pathways involved in tumorigenesis, have been intensely investigated. Disrupting the initiation and halting the progression of angiogenesis by targeting these biomarkers and molecular pathways has been considered as a potential treatment approach for tumor angiogenesis. This review discusses the currently known biomarkers and available antiangiogenic therapies in cancer, i.e., monoclonal antibodies, aptamers, small molecular inhibitors, miRNAs, siRNAs, angiostatin, endostatin, and melatonin analogues, either approved by the U.S. Food and Drug Administration or currently under clinical and preclinical investigations.

## 1. Introduction

Angiogenesis is a biological phenomenon, where new blood capillaries in adults are developed from preexisting primary blood vessels by sprouting and branching, responding directly to tissue demands [1].Vascularization is a prerequisite for fulfilling the increased demand for oxygen, nutrient supply to the growing cancer/tumor cells, and waste removal from the cells [2]. Chronic and sustained angiogenesis, a hallmark of cancer, is vital for continued tumor mass development, and is functionally essential for multistage tumorigenesis [3]. Interactions between the stimulatory, mediator, and regulator molecules regulate the proliferative and invasive activity of endothelial cells (ECs), resulting in a new vascular framework. Tumor cells secrete molecules that initiate the angiogenic process, however, the cells cannot express angiogenesis inhibitors to halt the process. The resulting new vessels allow tumor growth beyond the diffusion-limited maximum size. Tumor cells lie close to blood vessels; therefore, the chances of tumor cell dissemination from the tumor into the blood circulation are high, making them metastatic [4]. Thus, the tumor vasculature can be exploited as a therapeutic target in the cancer treatment.

One of the major strategies to kill the cancerous cells is hindering the blood supply to these cells. Hence, the identification of effective angiogenesis biomarkers is an essential step for treating diseases associated with pathological angiogenesis. The deregulation of biomarkers could be related to the initiation and progression of diseases and could be applied for prognosis, diagnosis, and therapeutic purposes. These biomarkers are involved in several molecular pathways associated with angiogenesis in cancer. Various agents, such as specific antibodies, aptamers, small interfering ribonucleic acids (siRNAs), and therapeutic agents, have been developed to target these biomarkers [5–7].

This review analyses the role of angiogenesis in cancer development and discusses the currently known angiogenic therapeutic biomarkers exploited in antiangiogenic therapy. Further, the available therapeutic strategies targeting the angiogenic biomarkers has also been described. The review also focuses on the recent novel research associated with angiogenesis biomarkers, available therapeutic choices, and future perspectives.

## 2. Developmental and Pathological Angiogenesis

**Figure 1:**
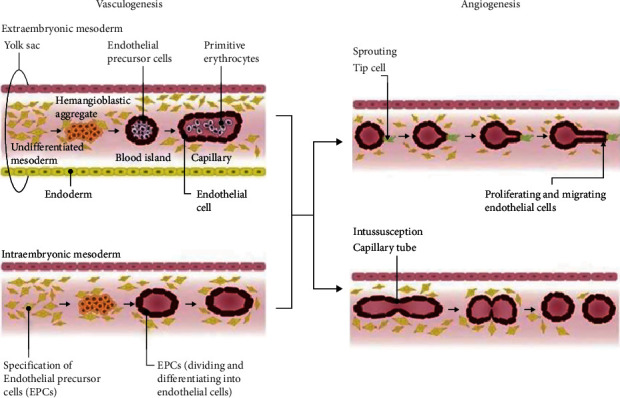
Diagrammatic presentation of vasculogenesis and angiogenesis, where hemangioblastic aggregates are formed from undifferentiated mesoderm, which further proceeds to endothelial precursor cells (packed in blood island) and primitive erythrocytes packed in layers of ECs. Also, tip cells sprout out of the proliferating and migrating ECs to form capillary tube [9].

Angiogenesis is a highly coordinated process involving series of complex events including proliferation and migration of ECs, vascular tube formation and anastomosis of new tubes, protease production and inclusion of smooth muscle cells [8]. Under normal physiological circumstances, novel ECs is generated and subsequently morph into tubes leading to angiogenesis. The *de novo* blood vessels formation during embryogenesis takes place via the event of vasculogenesis (Figure 1), in which angioblasts–primitive mesodermal cells subset form into primary blood vessel [8, 10].

**Figure 2:**
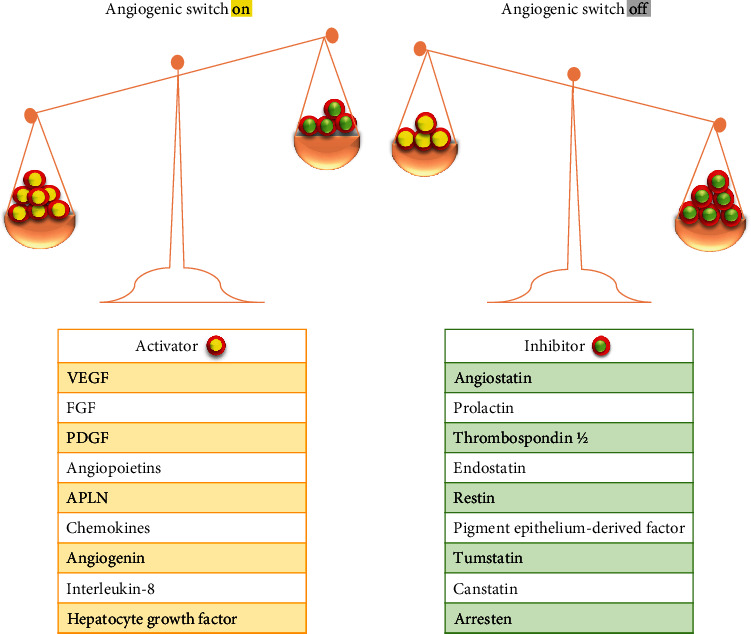
“Angiogenic switch” balance hypothesis. The angiogenic balance between angiogenic activators and inhibitors tightly regulates angiogenic switch mechanism. Upregulation of angiogenic inhibitors and angiogenic activators downregulation, spark angiogenesis leading to increased blood vessels formation. Reduction of inhibitor concentration i.e., angiostatin, restin, thrombospondin, and increasing the activator level i.e., vascular endothelial growth factor (VEGF); basic fibroblast growth factor (bFGF); placenta growth factor (PGF); interleukin-8 (IL-8) could induce the growth of novel blood vessels.

Besides vasculogenesis, balance is disrupted between pro- and antiangiogenic factors, where proangiogenic factors are prominent, this event is termed as “angiogenic switch,” which trigger angiogenesis and initiates tumor progression (Figure 2) [7]. Normally, angiogenesis is uncommon as ECs are nonproliferative and vasculatures are quiescent, except of ovaries and uterus where angiogenesis is required for the reproduction and embryogenesis [11]. Classification of angiogenesis includes physiological angiogenesis, observed in embryonic development, wound repair, endometrial hyperplasia during menstrual cycle, and pathological angiogenesis seen in tumors, diabetes, and chronic hepatitis [12]. Some of the examples of pathological conditions whose underlying cause is abnormal angiogenesis have been mentioned in Table 1.

Angiogenesis, a multistep process, is triggered by several biological signals, which direct the migration and differentiation of ECs [23]. The novel blood vessels formation is initiated *via* production of VEGF and other angiogenic factors in ECs, which then create wall of an existing small blood capillary, release the factors, and further bind to the surface receptors of ECs. Binding of these factors over ECs activate the series of signalling pathway, which triggers the secretion of enzymes i.e., matrix metalloproteinases (MMPs), followed by the degradation of the extracellular matrix (ECM) of the surrounding tissues and liberating sequestered growth factors from ECM. Further, invasion of the matrix, division and proliferation of ECs takes place. Finally, new ECs strings assemble into hollow tubes creating new network of blood vessels [24]. Recent studies have shown that the inhibition of angiogenesis is reported to be an important strategy to prevent multiple solid tumor, whereas enabling angiogenesis was proven to be critical for the success of tissue repair therapies [1, 24]. Hence, over the last two decades, several approaches have been deployed to target the angiogenic biomarkers.

The vessels growth in adults takes places via two fundamental processes: sprouting and intussusceptive angiogenesis, which occur in all tissues under specific physiological circumstances. Sprouting angiogenesis involves the origination of new capillaries from parental vessels by midvessel lateral budding. It involves (1) basement membrane degradation on the side of the dilated peritumoral post capillary lied close to the angiogenic stimulus; (2) ECs migration in connective tissue due to weakening of interendothelial contacts; (3) solid cord formation of ECs; (4) lumen formation takes place at the migrating front and functional capillary loops are established through anastomose of tubular sprouts, facilitated by synthesis of new basement membrane and pericytes recruitment [25]. Intussusceptive angiogenesis (nonsprouting angiogenesis) occurs when transluminal tissue develops within existing vessels without endothelial proliferation, and subsequently fuse to remodel the vascular plexus. It is a complementary method to sprouting angiogenesis and occurs in the zone of contact between two opposing capillary walls. The formation and fusion of transcapillary tissue pillars are the hallmark of intussusceptive angiogenesis where, longitudinal division of single capillary takes place forming two transluminal septa. ECs junction at opposing capillary walls form leaky bilayer, which allows the penetration of growth factors into the lumen [26]. The leaky contact zone filled with myofibroblasts and pericytes in order to build collagen fibers for vessels lumen development [27].

## 3. Role of Angiogenesis in the Cancer Pathogenesis

Angiogenesis is generally initiated from capillaries and its regulation exhibits a significant role in tumor progression and metastasis [28]. Malignant cells need consistent access of the circulatory system, hence tumor growth is accompanied by blood vessels ingrowth, either via new blood vessels formation or through co-optation of the preexistent vasculature [29]. As mentioned earlier, pro- and antiangiogenic factors regulate vascular homeostasis [30]. Vasculatures are quiescent and ECs are nonproliferative when these factors are balanced, while dominance of proangiogenic signalling initiates “angiogenic switch,” which activates the tumor growth from dormant state, sparking new blood vessels formation and a rapid growth of malignant cells [31].

Cancer cells, like normal body tissues requires adequate oxygen and metabolites supply and nourishment via vascular capillaries network [32]. Under normal conditions, ECs lining the interior surface do not multiply, restricting the capillaries proliferation. However, hypoxic (low levels of O_2_) and ischemic signals trigger various transcriptional responses and mediate the ECs precursor convergence, which give rise to capillary plexus and ultimately the development of the novel blood vessels [33]. Hypoxia is one of the physiological feature around tumor microenvironment, which occurs due to high oxygen concentration demands of uncontrolled proliferated cells for their aerobic metabolic activity [34]. Since oxygen demand exceeds the ability to supply through the preexisting blood vessels, tumor cells adapt this hypoxic condition by promoting angiogenic activity i.e., development of novel blood vessels from exiting one [35]. During the onset of tumor, angiogenesis is not stimulated, and its growth remain limited with low oxygen and nutrient supply. In early phase, cell proliferation counterbalances the cell death occurred due to hypoxic condition, and therefore tumor may dwell in dormant state. Angiogenesis is a critical prerequisite for the tumor progression beyond 1–2 mm^3^. Beyond which, hypoxic microenvironment around the growing tumors activates angiogenic network via upregulation of hypoxia-inducible transcription factor, which triggers various specific transcriptional responses such as cell division, metabolism, and angiogenesis [36]. Furthermore, angiogenic switch is activated by the tumor in the response of augmented angiogenic factors, resulting in the irreversible evolution of an active angiogenic state. Recruitment of new capillaries supplies oxygen and nutrients actively to angiogenic as well as nonangiogenic cells, leading to rapid tumor growth [37].

Upregulation of hypoxia-inducible transcription factor activates “angiogenesis” by activating oncogene. Oncogene activation expresses cytokines proangiogenic factors and suppresses antiangiogenic factors, which lead to the upregulation and uncontrolled angiogenic networking during tumor angiogenesis [38]. Angiogenesis is a coordinated regulation of these proangiogenic and antiangiogenic factors. VEGF, most potent proangiogenic factor and originally determined as vascular permeability factor, induces formation of blood vessels in tumors. Hypoxia instigates VEGF upregulation which is secreted by tumor cells during tumor angiogenesis. VEGF activates VEGF receptor-2 (VEGFR-2) expressed over ECs, which orchestrates the growth of blood vessels and induces EC proliferation [39]. Signalling pathway initiated via VEGFR-2 activation induces various endothelial responses including cell proliferation, vascular permeability, invasion, migration which is coupled with tumor progression and metastasis along with increased vessel density [40].

**Figure 3:**
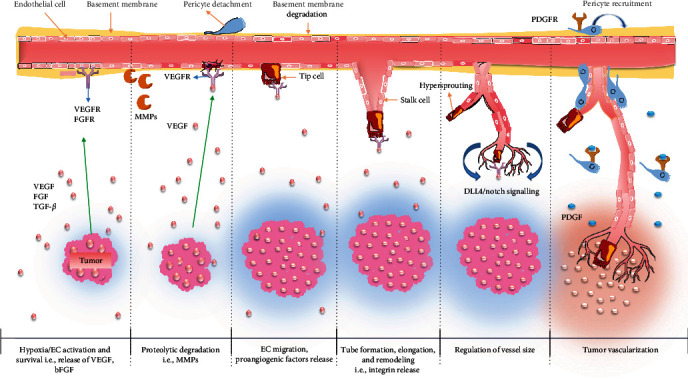
Mechanism of angiogenesis in cancer. Hypoxia induces the expression of hypoxia inducible factor (HIF), which consequently releases proangiogenic factors, such as VEGF, and upregulates the expression of protease, which leads to basement membrane degradation and pericytes detachment. Furthermore, specialized ECs migrate along the angiogenic factor gradient and differentiate into highly proliferative stalk cells, which initiate the formation of new vessels. PGDF stimulation promotes the attachment of pericytes with reduced proliferation and VEGF sensitivity. VEGF stimulates DLL4 secretion, which binds to Notch-1 receptors, downregulates VEGFR, and suppresses proliferation. Blood supply stimulates further tumor growth.

VEGF induces vascular permeability, which is considered as prerequisite for angiogenesis, via several mechanisms such as fenestrae induction, junctional remodeling, and vesiculo–vascular organelles. In addition to VEGF, MMPs induce angiogenesis via ECM degradation and ECs migration. Other important proangiogenic factors and their respective cognate receptors which promotes different stages of angiogenesis in tumor are bFGF, platelet-derived growth factor (PDGF), chemokines, ephrins, angiopoietins (ANGPTs), and apelin (APLN) [41, 42]. FGF-2 (or bFGF), a proangiogenic mediator, which acts together with VEGF and promote angiogenesis via inducing MMPs secretion and activates collagenase and plasminogen enzymes [43]. PDGF-B induces VEGF upregulation on tumor-associated ECs and pericytes recruitment in newly formed vessels [44]. ANGPTs are the growth factors, mediated through VEGF-independent pathways which promote angiogenesis via regulating blood vessels' remodeling and development [45]. Studies revealed that interplay of the growth factors—VEGF, MMP, and bFGF/FGF-2 promote active angiogenesis and tumor development.  Figure 3 represents the schematic diagram showing the role of angiogenic factors in tumor vascularization.

Tumor angiogenic vessels display unique features and are well-differentiated from normal blood vessels, thus provide an appealing targeting site for angiogenic therapeutics. The key differences are as follows: (a) genetic stability of ECs of tumor vasculature, thus the chances of developing resistance are low; (b) compared to normal vessels, tumor blood vessels are morphologically leakier, fenestrated, and possess higher vascular permeability, however tumor tissue has impaired lymphatic drainage which leads to the enhanced permeability and retention (EPR) causing more accumulation of nanotherapeutics at the tumor site; (c) proteomics and genomics-based studies indicated the expression of the specific biomarkers (receptors or antigens) at the ECs of tumor vasculature, which are present at insignificant levels in normal blood vessels. These biomarkers are associated with angiogenic processes and can be the proficient targeting sites for tumor therapeutics [46–50]

## 4. Prognostic and Therapeutic Angiogenic Biomarkers

A biomarker is a characteristic indicator of normal biological processes or pharmacological responses to a therapeutic intervention, which is measured objectively and evaluated. Biomarkers in “cancerous cells” can be detected in the patients, which may further define the prognosis and diagnosis of diseases. The predictive biomarkers can also be used to predict the therapeutic response in patient to the therapeutic agents and potential toxicity associated with the drug. Hence, “biomarkers” may further define the optimal therapeutic strategy for cancer patients, thus augmenting the therapeutic response and minimizing the therapy-related toxicity. The antiangiogenic therapy is an effective strategy for cancer treatment and identification of biomarkers for angiogenesis could be the future for development of antiangiogenic drugs. Various strategies have been explored for targeted delivery of these drugs [51, 52]. Generally, angiogenic biomarkers are involved with initiation, progression, and metastasis of cancer and targeting these biomarkers could modulate the angiogenesis in cancerous cells. Since then, various angiogenic biomarkers has been explored, where VEGF has been identified as the most potent biomarker to inhibit the tumor proliferation as it has been overexpressed in the tumor angiogenesis. Humanized monoclonal antibody bevacizumab, and the multi-tyrosine kinase inhibitors (TKIs) such as sunitinib and sorafenib have been developed to target angiogenic biomarker and proven as effective therapeutic strategy for cancer treatment [53]. Besides VEGF, other biomarkers such as FGF, PDGF, and nucleolin has also been explored to design the specific antibody to target cellular pathways related with the cancer angiogenesis.

**Figure 4:**
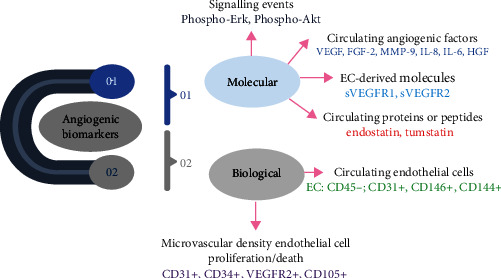
Major biomarkers for angiogenesis in preclinical and clinical studies.

Angiogenesis induction is considered to be one of the substantial hallmarks of cancer. The morphological distinctions between normal and angiogenic vessels have provided an insight regarding the normalization of cancer vasculature. However, antiangiogenic agents represent very complex mechanisms [54, 55]. Malignant cell genotypes manifest several physiological changes that explains the complications of cancer therapy. The tumor blood vasculatures show anomalous phenotypes i.e., immature morphological hierarchy, heterogeneous microenvironment, and highly permeable lumens, which arises due to the malfunction of ECs and their altered interaction with ECM. Also, the blood vessel compression due to the enhanced interstitial fluid in the cancer microenvironment modulates the mechanosensitivity of ECs with respect to the pressure gradient, which further generates the hypoxic and microenvironment with low pH leading to the cancer progression and production of ascites formation. The hypoxic environment further enhances the expression of angiogenic factors and proangiogenic activity of ECs. Also, these cells are highly susceptible to VEGF with significant upregulation of VEGFRs. Along with this VEGFRs, other angiogenic factors are also overexpressed, which makes the ECs more proliferative. Therefore, the identification of biomarkers could be an effective strategy for cancer treatment. Some of the major biomarkers for angiogenesis under clinical and preclinical studies are mentioned schematically in Figure 4 [56].

Targeting angiogenic biomarkers could reduce tumor mass and promote tumor regression, providing a rationale for antiangiogenic therapy for tumors. To date, several antiangiogenic treatments have been approved by the Food and Drug Administration (FDA), that target proangiogenic growth factors and their receptors (Table 2). Many pharmaceutical companies have expended massive efforts over angiogenesis therapies involving angiogenesis inhibition in oncology and ophthalmology, as well as angiogenesis stimulation in tissue engineering and wound healing.

Tumor progression and development are dependent on the process of angiogenesis. Since, secreted cytokines were reported to play a substantial role in angiogenesis by mediating tumors neovascularization, thus indicating their potential role as biomarker candidate for disease detection and treatment response [59]. Numerous angiogenesis markers have been reported till now that have represented simultaneous expression and effective cooperation at different stages of tumor angiogenesis [56]. Some important angiogenesis biomarkers explored for cancer therapy are discussed in the following subsection. Various proangiogenic factors that serve as potential biomarkers in cancer therapy are VEGF, bFGF, IL-8, PDGF, MMPs, endoglin, tissue factor, and hypoxia tissue factor [60–71] and among them, the important angiogenesis biomarkers explored for cancer therapy are discussed in the following subsection.

### 4.1. Vascular Endothelial Growth Factor

VEGF is a key regulator of physiological and pathological angiogenic events, and VEGF-A is the most widely known and major factor in tumor angiogenesis. VEGF/VEGFRs interaction is considered as a chief angiogenic regulator and dominant target for numerous antiangiogenic drugs [72]. The expression of VEGF is induced due to the hypoxic stimulus as a result of loss of tumor suppressor genes i.e., VHL and p53. VEGF are overexpressed in malignant tumors like breast, colorectal, lung, and prostate cancer. VEGF induces ECs proliferation via the ERK (extracellular signal-regulated kinase) and PI3K/Akt (phosphoinositide 3-kinases/protein kinases B) pathways. ECs migration downstream of VEGFR2 is induced through signalling pathway involving Rho GTPases and PI3K activation [73]. VEGF overexpression has been reported in solid tumors, therefore VEGF is considered as a potential marker for cancer [74].VEGF-A, angiogenic multifunctional mediator, binds to extracellular domain of VEGFR2 and transduce the responses of VEGF in ECs including ECs survival and proliferation, migration, permeability, and formation of capillary lumen, thus orchestrating the vasculature of cancer. Recent studies have suggested that VEGF stimulates the overexpression of myeloid cell leukaemia 1 (MCL-1) in cancers and malignancies, which is essential for cancer cell survival and development due to the balance disruption between anti- and proapoptotic proteins [75]. VEGF also interacts with angioregulatory immune cells and modulates T cells as well as myeloid cells in a VEGFR-mediated conduct. These immune cells release pro- or antiangiogenic agents via intercellular signalling and immune cells polarization to demonstrate inhibitory or modulatory characteristics, thus coordinating the cancer angiogenesis progression [76].

VEGF blockers inhibits tumor growth by preventing VEGFRs activation via neutralization of all bioactive forms of VEGF. However, patient may develop resistance to VEGF signalling pathway blockage by opting compensatory and adaptive mechanism through other mediators of angiogenesis such as PDGF or FGF [77, 78]. Therefore, blockage of VEGF signalling pathway via neutralizing antibodies to VEGF was reported to be ineffective as a monotherapy and occurrence of resistance was witnessed. VEGF activates PI3K/Akt/endothelial nitric oxide synthase signalling conduit, which stimulates ECs proliferation and vascular permeability. However, T cell-specific adaptor-c-Src signalling pathway is also involved in increasing the vascular permeability via separation of the endothelial junctions, which in turn is modulated via VEGF [79].

Various studies have proven the advantages of VEGF/VEGFR-based angiogenesis therapy. Recently, combination of VEGF-targeted angiogenic therapy and immune checkpoint inhibitors are under clinical trial, which are being conducted for melanoma, glioblastoma, and renal cancer therapy. Adaptive mechanisms that are responsible for resistance are: (a) upregulation of different proangiogenic factors; (b) alternative angiogenic signalling pathway activation; (c) vascular mimicry, a process in which cancer cells form blood vessels without involvement of ECs; (d) vascular co-option, in which tumor cells avoid angiogenesis via proliferating near existing blood vessels; (e) recruitment of endothelial progenitor cells; and (f) cell mobilization with a proangiogenic phenotype [81]. To improve the efficacy of antiangiogenic drugs, alternative angiogenic pathways need to be targeted along with the VEGF signalling pathway, or a combination of antiangiogenic therapy with chemo- or radiotherapy could be an effective solution to achieve optimal inhibition of cancer angiogenesis [82]. Several angiogenic agents such as aflibercept and ramucirumab targeting VEGF biomarker and VEGFR signalling pathway have been established till now.

Several antiangiogenic drugs based on VEGF/VEGFR signalling inhibition have been developed in the last decade. Multiple agents have been developed, including ribozymes, aptamers, soluble receptors, and small-molecule inhibitors, which aim to improve the efficacy, reduce toxicity, and optimize the clinical use of these therapies in combination with other therapeutic modalities. There is an urgent requirement for the identification of angiogenic therapeutic agents, optimal combination of therapeutic agent, doses and order of usage, and methods to monitor therapeutic results. Hence, research on the antiangiogenic agents targeting VEGF biomarkers holds immense potential for the advancement of cancer therapy.

### 4.2. Fibroblast Growth Factor (FGF)

FGFs belong to the family of heparin-binding growth factors, and exert their proangiogenic activity via interaction with ECs surface receptors, involving tyrosine kinase receptors, integrins, and heparan-sulphate proteoglycans [83]. FGF signalling regulates blood vascular development by activating ECs proliferation, migration, and sprouting. It modulates ECs metabolism responsible for ECM modulation [84]. FGF expression in tumors via activation of FGF signalling pathway, is utilized by tumor cells to escape VEGF-targeted therapies, inducing antiangiogenic therapeutic resistance. In preclinical studies, dual inhibitors targeting VEGF and FGF pathways simultaneously, have been proven efficacious against cancer.

FGFs are angiogenic biomarkers that are involved in the regulation of cell growth and differentiation, where FGFR-1 is expressed primarily over ECs and its overexpression is associated with cancer. The overexpression of FGF is associated with the various mutations, including gene amplification, altered gene splicing, etc., which could enhance the angiogenic process through stimulation and release of other proangiogenic factors. Studies have suggested that FGF acts in synergistic manner with VEGF to augment the tumor angiogenesis. Hence, the collaborative interaction between FGF and VEGF signalling has shown to be essential for the angiogenic processes; and targeting these pathways simultaneously could supress the angiogenesis more effectively as compared to targeting either pathway alone. In preclinical studies, dual inhibitors targeting the VEGF and FGF pathways have proven efficacious against cancer [85].

### 4.3. Platelet-Derived Growth Factor (PDGF)

PDGF signalling promotes the secretion of proangiogenic factor, which induces VEGF upregulation and enhances lymphatic angiogenesis along with the ECs proliferation and migration to form tube [86]. *In vitro* studies of human umbilical veins ECs treated with a PDGFR inhibitor and multi-tyrosine kinase inhibitors (TKI) showed reduced tube forming capacity of ECs [87]. Till now four PDGFs i.e., PDGF-A, PDGF-B, PDGF-C, and PDGF-D have been identified, where PDGF-B was reported to stimulate pericytes recruitment in newly formed blood vessels. Wang et al. examined PDGF-B and its receptor PDGFR expression in clear cell renal cell carcinoma (ccRCC) to evaluate the function of PDGF-B during angiogenesis. PDGF-B represented increased proliferation of vascular smooth muscle cells and migration capability during angiogenesis. Results suggested the proficiency of PDGF-B as promising prognostic marker [88]. Inhibition of PDGF-B signalling could commence vessel walls normalization and could be targeted along with VEGF signalling pathway for effective antiangiogenic therapy [89].

PDGFR has been regarded as a significant angiogenic factor, responsible for the expansion of metastatic tumors. It has been demonstrated as a major target for the TKI developed for cancer therapy. Recent studies suggested that interaction of PDGFR pathway with other signalling pathways (P13K/Akt, Ras-MAPK, JAK/STAT, and notch signalling pathway) could accelerate the cancer growth and reduce the sensitivity of cancerous cells. Various strategies have been explored till now to obstruct the PDGF pathway such as (i) usage of neutralizing antibodies or aptamers that may act as ligand traps; (ii) employing antibodies or small molecule inhibitors to disrupt the interaction between the ligand and receptor; or (iii) obstructing the PDGFR kinase function via low-molecular weight inhibitors [90]. Currently, Crenolanib besylate, a PDGFR inhibitor developed by AROG pharmaceuticals has shown to block the PDGFR phosphorylation and proven to be effective RTK inhibitors [91].

### 4.4. Angiopoietin (ANGPT)

The ANGPTs family comprises the two major ligands where ANGPT-1 promotes the maturation and stabilization of newly formed vessels via Akt/P13K pathway, while ANGPT-2 induces vessel destabilization and sprouting, detachment of pericytes and angiogenesis [92]. ANGPTs bind exclusively to Tie2 receptor tyrosine kinase [93]. ANGPT-2 expression is minimal in physiological conditions but is increased in response to VEGF and hypoxia in tumor-associated vessels [94]. ANGPT-2 upregulation in glioblastoma have been associated with increased resistance to therapy and reduced efficacy in anti-VEGF treatment [95]. Studies suggest that the inhibition of ANGPT-2 along with VEGFR-2 improved survival of glioma bearing mice by blocking macrophage recruitment, impairing tumor growth, and prolonging normalization of vessels. Therefore, ANGPT-2 and VEGFR-2 co-targeting could be effective in tumor therapy [96]. ANGPT1 is one of the ANGPTs, which regulates the integrity of ECs junction via accumulating factors such as vascular endothelial cadherin at the junction, where it permeates the proteins like VEGF and involved in the stabilization of actin cytoskeletons at the ECs junction [92].

Various ANGPT inhibitors are under clinical trials including but not limited to AMG 786 (Trebananib) and REGN 910 (Nesvacumab). Vanucizumab and RG7716 (Faricimab) served as dual inhibitor of ANGPT and VEGF have also demonstrated the enormous potential for cancer treatment. AMG 786, a peptide antibody, is one of the most effective therapeutic agent and nonspecific inhibitor of ANGPT-1 and ANGPT-2, while REGN 910, human monoclonal antibody binds specifically to ANGPT-2 and phase I clinical studies showed that it is efficacious and possess desirable safety profile [92].

### 4.5. Apelin (APLN)

Apelin receptor (APLNR) expression is restricted to the ECs of developing vascular system during the process of angiogenesis [97]. APLN expression stimulates microvascular proliferation inside tumors' cells and promote tumor development via enhancing angiogenesis, metastasis, and cancer stem cells development [98]. APL could indicate the diagnostic index for the degree of cancer progression, therefore it could serve as a potential biomarker for targeted therapy for cancers and pharmacological blockage via APLNR antagonists [99]. Moreover, APLN targeting could reduce tumor growth, improve blood vessels' function, reduces the invasiveness for tumor cells, and prevent resistance associated with angiogenic therapy [100, 101]. In the recent study, APLN was reported as an activator of the autophagy and showed to promote cell migration in lung carcinoma [102]. In a different study, targeting APLNR with an antagonist exhibited reduced tumor growth in mice [103]. Therefore, targeting APLN/APLNR signalling pathway could be a promising strategy to treat cancer.

The overexpression of APLN biomarker is coupled with the increased microvessel densities and cancer progression in various cancer including nonsmall cell lung cancer and hepatocellular cancer. APLN regulates the microvasculature proliferation and APLN antagonists (F13A and bevacizumab) showed the cancer progression inhibition via reducing this vascular density. Research suggested that APLN pathway has positive outcome on the cancer angiogenesis and disruption of this pathway could be effective for the antiangiogenic therapy in the therapeutic intervention of cancer [104].

### 4.6. Chemokines

Chemokines, members of the heparin-binding protein family, have emerged as important angiogenesis regulators and promote tumor angiogenesis either via binding through chemokine receptors expressed on ECs or through inflammatory cell recruitment. Chemokines regulate immune responses along with angiogenesis, conferring their dominant role in tissue microenvironment modulation; therefore, chemokines may serve as a potential biomarkers for targeting tumor angiogenesis [105]. Chemokine subfamily classifications based on the amount of cysteine residue deposition at the N-terminal domain of the molecules are CXC, CC, C, and CX3C. The CXC family is further classified based on the presence or absence of the ELR (glu–leu–arg) motif at their N-terminus and is thus indicated as ELR+ and ELR- chemokines, respectively [106]. ELR+ includes CXCL1, CXCL2, CXCL3, CXCL5, CXCL6, CXCL7, and CXCL8, which binds to the receptor CXCR2, that are overexpressed in microvascular ECs and tumor vessels, and enhances angiogenesis [107]. CXCL8 has been reported to induce release of VEGF and MMP-2, which are involved in metastasis-related tissue remodeling, along with the progression and cancer metastasis. Elevated CXCL8 serum level is associated with the severe tumor load and distant metastasis [108]. CL2 interacts with C–C chemokine receptor type 2, expressed in tumor endothelial progenitor cells and enhances endothelial permeability and metastasis. Thus, based on encouraging preclinical studies, cytokines could be explored as effective biomarkers for the establishment of antiangiogenic therapy.

## 5. Antiangiogenesis-Based Therapy for Cancer Treatment

**Figure 5:**
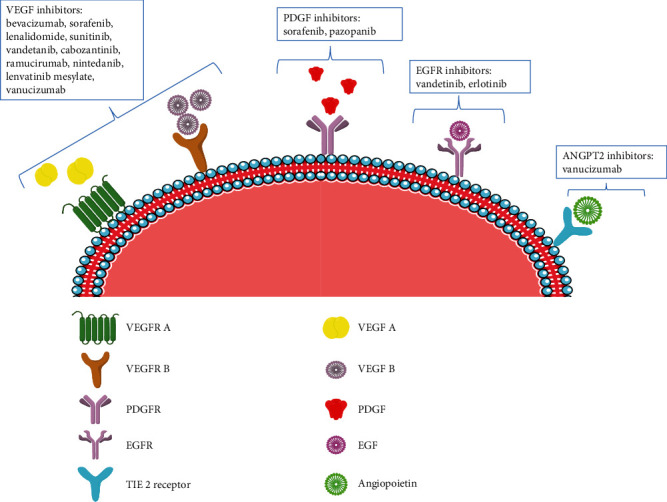
Antiangiogenic drugs and their targets such as VEGF, PDGF, EGF, and ANGPT2 along with biological substrates and respective inhibitors available in the market.

Antiangiogenic agents block the supply of oxygen and nutrients to cancerous cells. In 1971, Folkman hypothesized regarding the effectiveness of antiangiogenic agents for cancer therapy that these antiangiogenic agents could prevent the formation of new blood vessels and disrupt the existing one by neutralizing the angiogenic protein, inducing EC apoptosis, or inhibiting the endothelial receptors for angiogenic proteins [81]. These inhibitors are capable of targeting angiogenic growth factor receptors, Tei receptor, VEGFR, and PDGFR, or inhibit angiogenic growth factors, PGF and its receptor, VEGF, and bFGF [109]. Therefore, clinical strategies to develop molecules that target angiogenesis molecular pathways have been extensively researched for the treatment of cancers. As mentioned earlier, VEGF could be a potential biomarker, and various clinically available antiangiogenic agents act by targeting the VEGF/VEGFRs pathway, such as monoclonal antibodies (Bevacizumab), small-molecule TKI (Sorafenib), and VEGFR2 inhibitors (Ramucirumab), out of which monoclonal antibodies are being used widely, which act by binding to circulating VEGF (Figure 5). Aptamers, single-stranded DNA or RNA (15–100 nucleotide) ligands that bind specifically to a target molecule with higher affinity and minimal or no immunogenicity, have also been studied for antiangiogenic therapy [110]. Pegaptanib sodium was the first USFDA approved RNA aptamer, developed using systematic evolution of ligands by exponential enrichment methodology directed against a VEGF isoform, is a potent angiogenesis inhibitor [111].

Gene therapy is also being utilized in antiangiogenesis therapy, which involves the introduction of genetic materials to target cells to reprogram their activity. Gene therapy showed more effective penetration into tumors and less immunogenicity [112]. Antiangiogenic gene therapy aimed at prohibiting the formation of novel vessels and inactivating the preexisting blood vessels [113]. Recently, scientists developed the human soluble FMS-like tyrosine kinase receptor 1 (sFlt-1) encoding recombinant adeno-associated virus-2 (rAAV) vector for sustained antiangiogenic effect, without vector-associated immunity or toxicity [114]. The list of FDA approved angiogenesis inhibitors is mentioned in Table 2.

### 5.1. Monoclonal Antibodies (mAbs)

Monoclonal antibody-based therapy is an extensively explored strategy for targeting angiogenic biomarkers. Bevacizumab was the first FDA-approved humanized monoclonal antibody for the treatment of metastatic colorectal cancer in combination with chemotherapy that targets VEGF-A, which has been identified as a key factor for inducing tumor angiogenesis [115]. It is derived from murine VEGF, comprised of 93% human and 7% murine protein sequence and results of clinical trial demonstrated progression-free survival when combined with cytotoxic chemotherapies [116]. Currently, it is widely being used for tumor therapy; however monotherapy with bevacizumab may be insufficient for angiogenesis therapy as frequent resistance have been reported therefore generally prescribed in combination with the other chemotherapeutic agents [117, 118].

Ramucirumab, a USFDA approved human mAb has high selectivity for VEGFR-2, act via blocking the interaction between VEGF and its receptor [119]. Cetuximab, first USFDA approved monoclonal antibody that binds to extracellular domain of EGFR with higher affinity than the natural ligand, blocking the tyrosine kinase-dependent signal transduction pathway. Cetuximab exerts antitumor effect due to decreased production of MMPs and VEGF [120]. Aflibercept, another antiangiogenic-agent, is a fusion protein composed of a constant Fc human IgG domain in combination with the second Ig domain of VEGFR-1 and the third Ig domain of VEGFR-2. Aflibercept targets the VEGF pathway in combination with chemotherapy regimens in triple-negative breast cancer [121]. Antibody conjugated delivery systems have been explored by the researchers, which could serve as an efficient tool for cancerous cells' targeting where certain antigens are overexpressed and may attack the blood vessels feeding tumor [122].

### 5.2. MicroRNAs/Small Interfering RNAs

MicroRNAs and siRNAs have been found to be efficient modulators of genes that express angiogenic factors in an angiogenesis animal model [123]. miR-126 has been reported to have dual functions in pathological angiogenesis, where miR-126-5p overexpression promotes angiogenesis and miR-126-3p silencing inhibits it [124]. In addition, the expression level of oncogenic proteins was reported to be reduced by miR143/145, which binds to the mRNAs of VEGF, KRAS, and EGFR, representing a growth inhibitory effect [125]. The roles of miRNAs in angiogenesis in different tumor therapies are presented in Table 3.

**Figure 6:**
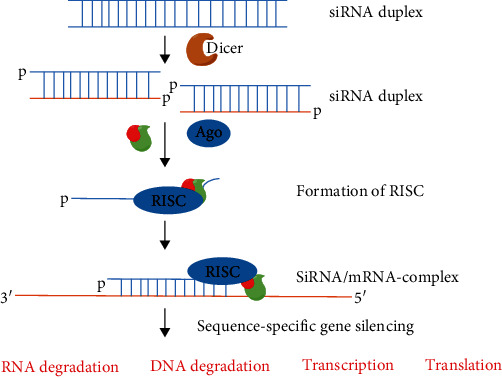
siRNA silencing pathway. Once inside the cytoplasm, siRNA is either directly incorporated into RNA-induced silencing complex (RISC) or undergoes a process mediated by Dicer. Upon RISC loading, the passenger strand dissociates, initiating the RNA interference process by cleaving and degrading the target mRNA.

KRAS mutations are responsible for the proliferation signalling of RAS/ ERK pathway and indicate poor response to EGFR inhibitors. Double-stranded RNA precursors are processed by a Dicer protein into short fragments, where one strand of the processed duplex is loaded into an argonaute protein (Ago), enabling RNA recognition and its expression modulation via several mechanism [210]. The pathway for siRNA silencing for a particular of gene is diagrammatically represented in Figure 6. Li et al. [211] developed multifunctional nanoparticles to improve VEGF gene silencing efficacy and improve tumor cell antiproliferation effects. The nanoparticles were coated with PEGylated histidine-grafted chitosan-lipoic acid and loaded with siVEGF and etoposide. The nanosystem utilizing siRNA have shown significant suppression of tumor growth and metastasis than monotherapy [211, 213, 214].

### 5.3. Small Molecular Inhibitors

Another strategy for targeting VEGF signalling involves TKI that targets VEGFR, such as Sunitinib, Pazopanib, and Axitinib (Table 2). TKIs target kinases, are being utilized more preferably as secondary and tertiary therapies and are reportedly more effective in combination with chemotherapy [215]. Axitinib was the first TKI compound with established antitumor activity that reduced vascular permeability, tumor volume, and tumor vascularization [212].

These angiogenesis inhibitors downregulate angiogenic activators that promote unregulated neovascularization in tumors. For example, affinitors (everolimus) and torisel (temsirolimus) downregulate angiogenesis by inhibiting the intracellular metabolic pathway of mTOR. Sorafenib is an FDA-approved TKI for hepatocellular carcinoma, metastatic thyroid carcinoma, and advanced RCC [216–218]. Withaferin A inhibits protein kinase C, which further inhibits apoptosis induction by caspase-3 activation and exhibits antiangiogenic activity [219]. Regorafenib is a multikinase inhibitor that restricts the kinases involved in tumor angiogenesis and oncogenesis (KIT, RET, RAF1, and BRAF), enhancing the survival of cancer patients [220].

### 5.4. Angiostatin and Endostatin

Endostatin blocks the binding of VEGF to ECs and inhibits the growth and migration of ECs followed by the suppression of capillary formation. Retinostat®, a Lentiviral Equine infectious anaemia virus vector-based therapy, was investigated for safety and tolerability in a Phase I clinical trial. The recombinant EIAV-based vector contains cDNAs expressing endostatin and angiostatin for long-term antiangiogenic activity in patients with macular degeneration [116, 117]. Angiostatin blocks matrix-enhanced plasminogen activation and inhibits cancer metastasis and invasion; however, angiostatin has a short t_1/2_, representing the requirement of a specialized delivery system. Zhang et al. [221], hypothesized that the combination of bevacizumab and angiostatin via attacking two different angiogenic pathways could lead to an additive antiangiogenic effect. The combination was tested in thymic mice bearing intracranial human glioma (U87), where the injection of G47*δ*-mAngio (an oncolytic virus expressing angiotensin) allowed bevacizumab-induced inhibition of invasion markers (MMP2 and MMP9) and angiostatin-mediated inhibition of VEGF expression. The results showed the enhanced antiangiogenic activity of a combination system utilizing viral oncolytic therapy [221].

Despite the development of several antiangiogenic agents, enormous challenges persist with respect to their efficacy, toxicity, drug resistance, and selection of patients who will benefit from antiangiogenic therapy. VEGF-targeted therapies are relatively safe, and several clinical trials have revealed several side effects that can be managed through proper care [222]. Despite the development of several antiangiogenic agents, enormous challenges persist with respect to their efficacy, toxicity, drug resistance, and selection of patients who will benefit from antiangiogenic therapy. VEGF-targeted therapies are relatively safe, and several clinical trials have revealed the side effects, which can be managed through proper care [223].

### 5.5. Melatonin and Its Analogues

The pharmacological potential of melatonin is found in various biological processes, including circadian rhythm synchronization, immune response stimulation, antioxidant activity, antiestrogen activity, and oncostatic activity. In addition, melatonin exhibits antiangiogenic activity in various cancers through multiple mechanisms, inhibiting cancer growth and metastasis [224]. Melatonin favors angiogenesis in some physiological events, skin lesions, and gastric ulcers while suppressing neovascularization in tissues in hypoxic environments (tumors) and age-associated eye disorders [225]. It also inhibits HIF-1-induced angiogenesis and thereby exerts antitumor action [226].

Melatonin-treated gastric tumor-bearing mice showed significantly reduced expression of both mRNA and protein levels of HIF-1*α*, RZR-ROR*γ*, and VEGF compared to untreated mice. These changes are attributed to melatonin's antiangiogenic potential in human gastric cancer cells [227]. It exhibits antiangiogenic activity by downregulating VEGFR-2 in ER-negative breast cancers [228]. Furthermore, no significant HIF-1*α* expression was observed in melatonin-treated tumors than in the vehicle control group. In contrast, melatonin significantly downregulated HIF1-*α* and VEGF expression in the liver and mouse tumor models [229]. In prostate cancer, melatonin promotes HIF-1*α* accumulation by suppressing ROS production and the sphingosine kinase-1 pathway, exhibiting antitumor action [230]. Melatonin treatment also resulted in a parallel reduction of VEGF, VEGFR-2, and HIF-1*α* expression with tumor size and blood capillary density in ovarian tumor-carrying rats [231]. It also impairs vasculogenesis in oral cancer by inhibiting ROS-activated Akt and ERK signalling through the HIF-1*α* pathway and represses the expression of ROCK-1, HIF-1*α*, and VEGF genes in oral cancer [232, 233].

Thus, the different mechanisms through which melatonin exhibits antiangiogenic activity are: (a) inhibition of HIF-1*α* translocation into the nucleus and downregulates the mRNA and proteins such as VEGF, phosphor-STAT3, and the CBP/p300 complex (referred to as angiogenesis-related gene expression); (b) inhibition of VEGF-induced VEGFR2 phosphorylation, thus suppressing the expression and transactivation of VEGFR2; and (c) inhibition of the migration and invasion of ECs in tumor tissues; (d) melatonin receptor (especially MT1) mediated downregulation of VEGF in some cancers [234]; however, receptor involvement in the downregulation of VEGF was independent in tumor tissues and melatonin possesses antiangiogenic effects in tumor tissues, making melatonin, and its analogues a potentially promising drug to inhibit tumor growth and metastasis [235–237].

Hence, melatonin and its analogues have gained the attention of new researchers to evaluate their potential as an anticancer drug either as an adjuvant or as a novel formulation in combination with standard anticancer drugs. The synthetic analogues of melatonin, agomelatine and ramelteon can be explored for their anticancer potential against various cancers through their mechanism of inhibiting angiogenesis and the epithelial mesenchymal transition pathway [238].

## 6. Challenges and Future Direction of Antiangiogenic Therapy

One of the major challenges associated with the antiangiogenic therapy is the heterogeneous nature of cancer. Since angiogenesis is a natural physiological phenomenon that should be maintained for the proper balance for haemostasis, therefore the identification of specific biomarkers is required to avoid damage to healthy organ. Currently, the identification of prognostic biomarkers is the promising strategy for the development of antiangiogenesis therapy. However, modulating the process of angiogenesis via recognized biomarker requires profound insight regarding the molecular mechanisms through which angiogenesis is mediated. Also, resistance mechanisms of antiangiogenic agents can be revealed via the bioprofile information, which can further disclose the additional mechanisms for angiogenesis that can be targeted for cancer therapy. Currently, out of all the available antiangiogenic agents, none has met the expectations regarding the survival of cancer patients. The identification of angiogenic biomarkers and its application in cancer therapy, has been the main objective and vision yet to achieve. Various inhibitors of angiogenic markers including monoclonal antibodies have performed well in specific, but not all, cancers. Hence, extensive research is going on to endorse the better understanding of compensatory pathway within tumor cells and develop the agents with therapeutic potential to inhibit the angiogenesis in cancer.

## 7. Conclusion

The crucial role of angiogenesis in pathological alterations, especially in cancer progression, proliferation, and metastasis, and how it keeps a regulatory eye on other remaining hallmarks of cancer are extensively detailed in this manuscript. The functioning of prognostic and angiogenic biomarkers like VEGF, FGF, PDGF, ANGPTs, APLN, and chemokines interplay in mediating the progression of angiogenesis are detailed. The antiangiogenic therapy, including monoclonal antibodies, siRNAs, miRNAs, small molecule inhibitors, angiostatin, endostatin, and melatonin analogues, functions in inhibiting angiogenesis through altering angiogenic biomarkers' expression are also described here. However, numerous challenges are on the way for miRNAs and siRNAs to endorse them at the clinical level due to the avoidance of acceptance by human society for treatment and management of disease using foreign genetic materials. Also, the single miRNAs and siRNAs have been incapable of defeating the intensified stages and multiple pathways supporting angiogenesis in various cancer stages. The multicomponent formulations could be possible for sequential blocking of angiogenesis, and transforming the same at the clinical level seems impossible with numerous challenges. Moreover, designing a novel melatonin receptor subtype 1 could be an antiangiogenic candidate for targeting cancer. The anti-HIF-1*α* phytochemicals can also be explored for inhibiting angiogenesis innervating the tumor tissues. The more network-based studies and artificial intelligence processing are needed to explore these possible agents to target angiogenic pathways for the cancer treatment.

## Figures and Tables

**Table 1:** Diseases associated with impaired angiogenesis.

Diseases in human	Organ/tissue	References
Cancer, systemic sclerosis	Multiorgan	[13, 14]
Diabetes, atherosclerotic plaque	Cardiovascular system	[15, 16]
Multiple sclerosis	Nervous system	[17]
Inflammatory bowel diseases	GIT	[18]
Psoriasis	Skin	[19]
Endometriosis	Reproductive system	[20]
Obesity	Adipose	[21]
Asthma	Respiratory system	[22]

**Table 2:** FDA approved antiangiogenic agents for cancer therapeutics [41, 57, 58].

Antiangiogenic agent	Brand name	Company	Target biomarker molecule	Disease indication
*Monoclonal antibodies*				
Ramucirumab	Cyramza®	Eli Lily	VEGFR-2	Metastatic nonsmall cell lung carcinoma, gastric cancer, colorectal cancer
Bevacizumab	Avastin®	Genentech/Roche	VEGFR	Metastatic colorectal cancer, nonsmall cell lungs cancer, cRCC, ovarian cancer, metastatic breast cancer, glioblastoma
Cetuximab	Erbitux®	Bristol-myers squibb	EGFR	Second line treatment for colorectal cancer, squamous cell carcinoma of head and neck
Panitumumab	Vectibix®	Amgen	EGFR	Colorectal cancer
Necitumumab	Portrazza	Eli Lily	EGFR	Squamous nonsmall-cell cancer

*TKI*				
Axitinib	Inlyta®	Pfizer	VEGFR_1–3_ and PDGFR	Metastatic hepatocellular cancer (HCC), thyroid cancer, renal cell carcinoma
Imatinib mesylate	Gleevec®	Novartis	Blocks Abelson cytoplasmic tyrokinase and PDGFR activity	Chronic myeloid leukaemia, gastrointestinal stromal tumors, myelodysplastic, myeloproliferative disease
Nintedanib	Vargatef®	Boehringer ingelheim	VEGFR, PDGFR, FGFR	Idiopathic pulmonary fibrosis, nonsmall cell lung cancer
Sunitinib malate	Sutent®	Pfizer	VEGFR_1–3_, PDGFR	Pancreatic cancer, RCC, gastrointestinal stroma tumor
Pazopanib	Votrient®	Novartis	VEGFR_1–3_, PDGFR	Metastatic renal cell cancer, advanced soft tissue sarcoma
Vandetanib	Caprelsa®	Sanofi	EGFR, VEGFR_2–3_, PDGFR	Pancreatic cancer, advanced metastatic renal cell cancer
Sorafenib	Nexavar®	Bayer/Onyx pharmaceuticals	VEGFR_1–3_, PDGFR*β*, RET	Hepatocellular cancer, iodine resistant advanced thyroid carcinoma
Regorafenib	–	–	VEGFR_1–3_, TIE2	Metastatic colorectal cancer, hepatocellular carcinoma, gastrointestinal stroma tumor

*Receptor fusion protein*				
Aflibercept	Zaltrap®	Regeneron pharmaceuticals	VEGF A and B	Metastatic colorectal cancer

Aptamers				
Pegaptanib sodium (NX1838)	Macugen®	Eyetech.IN/Pfizer	VEGF-165	Macular degeneration

**Table 3:** Role of microRNAs against angiogenesis in different tumor therapy.

S. no.	microRNA	Gene targets	References
*Breast cancer*			

1.	miR-126	PI3K regulatory subunit 2; VEGF antisense; cluster of differentiation 97; insulin-like growth factor binding protein 2	[126–129]

2.	miR-21 miR-497	HIF-1 *α*; VEGFR2	[130–132]

3.	miR-155	von Hippel-Lindau	[133]
	miR-199b-5p	Activin receptor-like kinase-1-downregulation; attenuated ALK1/Smad/Id1 pathway	[134]

4.	miR-57 miR-573	VEGF antisense, focal adhesion kinase, ANGPT2, HIF-1 *α*	[135]
	miR-204	ANGPT1, transforming growth factor beta receptor 2, phosphoinositide 3-kinases, Src	[136, 137]

5.	miR-542-3p	ANGPT2; CCAAT/enhancer-binding protein *β*; POU class 2 homeobox 1	[138–140]

7.	miR-4306	SIX1/Cdc42/VEGF antisense- downregulation; suppressed cell proliferation, migration and invasion and abrogates angiogenesis	[141]

*Pancreatic cancer*			

8.	miR-21 miR-199	HIF-1*α*; VEGF	[142, 143]

9.	miR-34a	Sirtuin 1 (SIRT1)	[144]

*Lung cancer*			

11.	miR-181d-5p	Cyclin dependent kinase inhibitor 3-downregulation; suppressed proliferation, and epithelial-mesenchymal transition, and increased cell apoptosis	[145]

12.	miR-126 let-7b	VEGFA	[146]

13.	miR-128	Serum VEGFC	[147]

14.	miR-195	VEGF- downregulation; suppressed the viability and migration and angiogenesis	[148]

15.	miR-494	Phosphatase and tensin homolog (PTEN)	[149]

16.	miR-210	VEGFR type 2	[150]

17.	miR-29c	PVT1-upregulation-promote VEGF pathway	[151]

*Colorectal cancer*			

18.	miR-126	VEGF, VEGFR2	[152, 153]

19.	miR-21	PTEN; tissue inhibitor of metalloproteinases-1 and 3 (TIMP1 and TIMP3)	[154]

20.	miR-30	Delta-like 4 (DLL4)	[155]

21.	miR-18a miR-19	Early growth response 1	[156]

22	miR-194	Tumor suppressor p53	[157, 158]

23.	miR-15-16	FGF2, and cyclin B1 (CCNB1)	[159, 160]

24.	miR-29b	FGF2, transcription factor 7-like 2 (TCF7L2), drosophila embryonic protein SNAI1 (SNAIL), B-cell CLL lymphoma 9-like protein (BCL9L), MMP2, and T-cell lymphoma invasion and metastasis (TIAM1)	[161–163]

25.	miR-27a miR-27b	DLL4, SPRY2, VEGFC, SGPP1, SMAD2	[164–167]

26.	miR-192	*β*-Cell lymphoma-2, ZEB2, VEGFA	[168]

27.	miR-145	AKT, N-RAS, IRS1, VEGF, p70S6K1	[169, 170]

28.	miR-143	AKT, HIF-1*α*, VEGF	[171]

29.	miR-23b	7FZD7, MAP3K1	[172]

30.	miR-1249	VEGF A/HMGA2-downregulation; suppressed colorectal cancer cell proliferation, migration, invasion, and angiogenesis, regulate Akt/mTOR pathway and EMT	[173]

*Ovarian cancer*			

31.	miR-199a miR-125b miR-145	HIF-1*α*, VEGF, p70S6K	[174]

32.	miR-484 miR-642 miR-217 miR-27a	VEGF, VEGFR2, COX2, SP1	[175, 176]

33.	miR-200 family	ZEB1, ZEB2, IL8, CXCL1	[177–179]

34.	miR-204	Inhibits brain-derived neurotrophic factor (BDNF)	[180]

35.	miR-765	miR-765 downregulates VEGFA/Akt1/SRC-*α* axis in SKOV3 (ovarian cancer cells)	[181]

*Gastric cancer*			

36.	miR-20	Downregulate VEGF and inhibits angiogenesis	[182]
37.	miR-29b		[183]
38.	miR-93		[184]
39.	miR-126		[185]
40.	miR-190		[186]
41.	miR-195		[187]
42.	miR-200		[188]
43.	miR-203		[189]
44.	miR-497		[190]
45.	miR-503		[191]
46.	miR-638		[192]

47.	miR-22	Targets VEGF inducers and regulates VEGF dependent angiogenesis	[193]
48.	miR-107		[194]
49.	miR-519c		[195]
50.	miR-145		[196]

51.	miR-616-3p	miR-616-3p upregulates VEGF-A/VEGFR2 and induce tumor angiogenesis	[197]

52.	miR-126	Directly inhibits VEGF-a expression and thereby inhibit angiogenesis both *in vitro* and *in vivo*	[198–200]

53.	miR-29a/c	Suppresses VEGF expression in GC cells, inhibiting cell growth, migration and angiogenesis	[201]

54.	miR-27b miR-101 miR-128	Downregulate VEGFC and thereby inhibit angiogenesis	[202]

55.	miR-590	Targets VEGF1/2 and NRP1 expression; inhibit migration, invasion and angiogenesis of GC both *in vivo* and *in vitro*	[203]

56.	miR-574-5p	Activates mitogen-activated protein kinases (MAPKs) through suppressing target gene, PTPN3 expression and promotes angiogenesis via enhancing VEGF-A expression	[204]

57.	miR-210	Highly expressed miRNA in hypoxic conditions and mediates metabolism, angiogenesis, and apoptosis	[205]

58.	miR-718 miR-382	Targets PTEN and thereby inhibits angiogenesis of gastric cancer	[206]

59.	miR-135b	Suppress FOXO1 protein and enhance angiogenesis in gastric cancer	[207]

*Glioma*			

60.	miR-26a	Overexpression inhibit PTEN and regulate angiogenesis	[208]

61.	miR-103a-3p miR-382-5p	miR-103a-3p and miR-382-5p overexpression activates PI3K/Akt signalling pathway and leads to upregulation of MOV10, circ-DICER1, ZIC4, and Hsp90*β* proteins which promotes cell viability, migration, and tube formation of glioma-exposed ECs	[209]

*Hepatocellular carcinoma*			

62.	miR-885-5p	Overexpression of miR-885-5p silences astrocyte elevated gene 1 (AEG1); inhibit EMT and angiogenesis	[210]

*Head and neck cancer*			

63.	miR-30e-5p	Overexpression of miR-30e-5p silences AEG1 suppresses migration o HUVECs and downregulation of VEGF and HGF, which leads to angiogenesis and metastasis	[211]

*Gall bladder cancer*			

64.	miR-136	Overexpression of miR-136 downregulates MAP2K4 ad inhibits angiogenesis and proliferation	[210]

*Renal cell carcinoma*			

65.	miR-21	miR-21 expression targets programed cell death protein 4 (PDCD4)/c-Jun signalling pathway and promotes the migration, invasion and angiogenesis in renal cell cancer cells	[212]

## Data Availability

The authors confirm that all the data supporting the findings of this study are presented within the article. If any further information is required, then it may be provided upon reasonable request.

## Abbreviations

ANGPTs: Angiopoietins
APLN: Apelin
APLNR: Apelin receptor
bFGF: Basic fibroblast growth factor
ECs: Endothelial cells
ECM: Extracellular matrix
ERK: Extracellular signal-regulated kinase
FGF-2: Fibroblast growth factor 2
HIF: Hypoxia inducible factor
IL-8: Interleukin-8
MMPs: Matrix metalloproteinases
PDGF: Platelet-derived growth factor
PDGFR: Platelet-derived growth factor receptor
PI3K/Akt: Phosphoinositide 3-kinases/protein kinase B
PTEN: Phosphatase and tensin homolog
RCC: Renal cell carcinoma
RISCs: RNA-induced silencing complex
TKI: Tyrosine kinase inhibitors
VEGF: Vascular endothelial growth factor
VEGFA: VEGF antisense
VEGFR: Vascular endothelial growth factor receptor.
